# The ELAVL1‐PLAUR‐suPAR Axis Exacerbates Diabetic Nephropathy by Promoting Podocyte Injury and Inflammation

**DOI:** 10.1111/1753-0407.70254

**Published:** 2026-08-05

**Authors:** DanDan Xu, Liang Xu, LinLin Li

**Affiliations:** ^1^ Department of Nephrology Henan Provincial People's Hospital Zhengzhou City Henan Province China

**Keywords:** diabetic nephropathy, ELAVL1, PLAUR, podocyte injury, suPAR

## Abstract

**Background:**

Diabetic nephropathy (DN) is a leading cause of end‐stage renal disease. ELAVL1, an RNA‐binding protein, is involved in the regulation of genes closely associated with DN pathogenesis, whereas suPAR acts as a circulating mediator that promotes podocyte damage and contributes to proteinuric kidney disorders. However, whether ELAVL1 regulates suPAR release in DN remains unexplored.

**Methods:**

Clinical samples from patients with DN were collected and analyzed. In vitro, podocytes were treated with high glucose, and ELAVL1 or PLAUR (encoding uPAR) was knocked down or overexpressed using lentiviral transduction. In db/db mice, podocyte‐specific manipulation of Elavl1 or Plaur was performed via adeno‐associated virus‐mediated delivery. Renal function was assessed by measuring the urinary albumin‐to‐creatinine ratio (UACR) and serum creatinine. Glomerular pathology and inflammatory markers were evaluated histologically and biochemically.

**Results:**

ELAVL1 was upregulated in DN and positively correlated with podocyte injury. Knockdown of ELAVL1 mitigated hyperglycemia‐induced podocyte damage and inflammatory responses. Mechanistically, ELAVL1 directly bound to and stabilized PLAUR mRNA, leading to increased uPAR protein expression and elevated suPAR secretion. The protective effects of ELAVL1 knockdown were reversed by PLAUR overexpression in podocytes. In diabetic mice, podocyte‐specific knockdown of Elavl1 or Plaur markedly lowered circulating suPAR levels and alleviated renal dysfunction and histopathological injury. Moreover, restoration of the PLAUR‐suPAR axis abolished the renoprotective benefits conferred by Elavl1 knockdown.

**Conclusion:**

Hyperglycemia‐induced ELAVL1 upregulates PLAUR and suPAR release from podocytes, promoting renal inflammation and injury. Targeting the ELAVL1‐PLAUR‐suPAR axis may offer a therapeutic strategy for DN.

## Highlights

- ELAVL1 promotes suPAR release in podocytes by directly binding to and stabilizing PLAUR mRNA.
- PLAUR‐suPAR mediates the protective effects of ELAVL1 knockdown in HG‐induced podocytes.
- Podocyte‐specific knockdown of Elavl1 or Plaur alleviates diabetic nephropathy progression in db/db mice.

## Graphical abstract

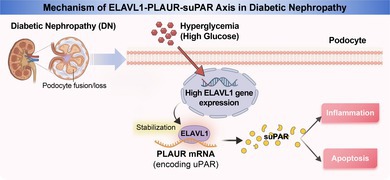

## 1 Introduction

Diabetic nephropathy (DN) is a severe diabetes‐related microvascular complication and remains a major contributor to end‐stage renal disease (ESRD) [1, 2]. Pathologically, it manifests as glomerular enlargement, thickening of the basement membrane, and expansion of the mesangial matrix, accompanied by progressively worsening proteinuria, which can ultimately lead to glomerulosclerosis [3]. Although maintaining blood glucose and blood pressure within target ranges remains the cornerstone of therapeutic intervention, a considerable proportion of patients continue to exhibit progressive renal deterioration. This observation indicates that critical molecular pathways contributing to the initiation and advancement of DN have yet to be fully defined. The multifaceted and intricate nature of DN pathogenesis therefore presents substantial challenges for effective treatment development. Apart from angiotensin‐converting enzyme inhibitors (ACEIs) and angiotensin receptor blockers (ARBs), which have been in use for over two decades, no other effective drugs for DN have been developed [4, 5]. Moreover, existing therapies, while beneficial, are limited by side effects such as hyperkalemia and hypotension, as well as long‐term issues like aldosterone escape, which can reduce treatment efficacy [6, 7]. Thus, there is a pressing need to identify more effective therapeutic targets for DN.

The development of DN arises from a complex interplay of multiple factors, including metabolic disturbances induced by hyperglycemia, hemodynamic changes, inflammatory responses, oxidative stress, and genetic susceptibility. These processes lead to glomerular hyperfiltration and hypertrophy, ultimately resulting in renal parenchymal fibrosis and scarring [8]. Podocytes are highly differentiated terminal cells whose unique foot processes and the slit diaphragms between them form the core of this filtration barrier [9]. Podocytes are pivotal cells essential for maintaining the integrity and function of the glomerular filtration barrier. Substantial evidence indicates that podocyte injury and loss are central to the early development of proteinuria and the decline in glomerular filtration function in DN [10, 11]. Consequently, elucidating the molecular mechanisms responsible for podocyte damage and pinpointing potential therapeutic targets is critically important for devising effective strategies to prevent and manage DN.

Over the past few years, considerable attention has been directed toward the soluble form of the Urokinase Plasminogen Activator Receptor (suPAR) as a circulating biomarker in the bloodstream. Accumulating evidence indicates that increased suPAR concentrations are strongly linked to the progression and adverse outcomes of several kidney disorders, including acute kidney injury (AKI), focal segmental glomerulosclerosis (FSGS), and DN [12, 13, 14]. Its pathogenic mechanism is believed to involve activating β3 integrin on the podocyte surface, disrupting cytoskeletal dynamics, leading to foot process effacement and proteinuria [15]. Research by Monte et al. [16] confirmed that blocking the uPAR pathway in a streptozotocin‐induced model ameliorated diabetic kidney injury, suggesting the uPA/uPAR system as a potential target for developing novel uPAR‐targeted therapies. However, within the context of DN, the upstream regulatory network driving suPAR production and release remains unclear, which hampers therapeutic development targeting this pathway.

Embryonic lethal abnormal visual‐like protein 1/human antigen R (ELAVL1/HuR) is an important RNA‐binding protein that post‐transcriptionally regulates the stability and translation efficiency of target mRNAs by binding to AU‐rich elements in their 3′‐untranslated regions (3′‐UTRs) [17]. ELAVL1 expression is upregulated in various inflammatory and fibrotic diseases and is involved in processes such as cellular stress, inflammatory responses, and apoptosis [18]. Studies on DN indicate that ELAVL1 contributes to high glucose‐induced inflammasome activation and fibrotic changes in renal tubular epithelial cells and mesangial cells [19, 20]. Beyond these cell types, ELAVL1 also exerts important functions in podocytes. For instance, Guo et al. [21] reported that ELAVL1 is dysregulated in DN via a GSK‐3β‐dependent mechanism and critically contributes to podocyte injury through post‐transcriptional regulation of multiple genes. Further evidence suggests that a feedback loop involving lncRNA 254 693 and ELAVL1 may participate in podocyte injury during DN pathogenesis [22]. Pharmacological inhibition of ELAVL1 with niclosamide has been shown to ameliorate hyperglycemia, renal inflammation, and oxidative stress in DN, underscoring its therapeutic relevance [23]. Additionally, targeting the ELAVL1‐acyl‐coenzyme A synthetase long‐chain family member 4 (ACSL4) axis can attenuate high glucose‐induced podocyte injury and ferroptosis, revealing another potential treatment strategy [24]. Most recently, ELAVL1 was found to aggravate podocyte injury, inflammation, and cell death by stabilizing ubiquitin‐specific peptidase 22 through an ACSL4‐dependent deubiquitination mechanism, thereby accelerating DN progression [25]. Despite these insights, it remains unclear whether ELAVL1 influences DN progression by regulating suPAR production in podocytes.

Based on the above background, we propose the following scientific hypothesis: In the diabetic kidney environment, factors such as high glucose induce the upregulation of ELAVL1 expression in podocytes. The upregulated ELAVL1 then directly binds to and stabilizes the mRNA of its downstream target, the uPAR (encoded by the PLAUR gene), thereby increasing uPAR protein expression and the secretion of its soluble form, suPAR. The released suPAR acts on podocytes in an autocrine or paracrine manner, exacerbating their injury, inflammation, and dysfunction, ultimately driving the pathological progression of DN. To test this hypothesis, this study integrates clinical sample analysis, in vitro cell experiments, and in vivo animal models. First, we confirmed alterations in the ELAVL1/PLAUR/suPAR axis and its correlation with renal injury in renal tissues from DN patients and in high glucose‐induced podocytes. Subsequently, using genetic approaches in podocytes, we demonstrated that ELAVL1 promotes suPAR release through stabilization of PLAUR mRNA, thereby leading to podocyte injury in vitro. This finding was further confirmed by rigorous rescue experiments. Finally, through podocyte‐specific knockdown in db/db mice combined with rigorous in vivo rescue experiments, we conclusively demonstrated the pathogenic role of this signaling axis and its therapeutic promise. Overall, this study reveals a previously unrecognized mechanism contributing to podocyte injury in DN, thereby providing a revised mechanistic understanding and pinpointing candidate targets for future therapy.

## 2 Methods

### 2.1 Clinical Sample Collection

Fasting morning venous blood and midstream urine samples were collected from 23 patients with DN and 23 age‐ and sex‐matched healthy controls without a history of diabetes, hypertension, or renal disease. Renal tissue samples from the DN patients were obtained via percutaneous needle biopsy for diagnostic purposes. The baseline demographic and clinical characteristics of these participants were recorded and are summarized in Table 1. In addition, 15 normal control renal tissue samples were acquired from wedge‐shaped resection specimens of patients undergoing nephrectomy for tumors; these samples were pathologically confirmed to consist of histologically normal renal parenchyma. The baseline demographic and clinical data of these tissue donors were also documented (Table S1).

**TABLE 1:** Baseline demographic and clinical characteristics of the serum/urine cohort.

Variable	Healthy controls (*n* = 23)	DN patients (*n* = 23)	*p*
Age, years	56.2 ± 9.6	57.8 ± 10.1	0.585
Male, *n* (%)	14 (60.9)	13 (56.5)	0.765
Diabetes duration, years	—	8.4 ± 5.0	—
HbA1c, %	5.2 ± 0.3	7.8 ± 1.5	< 0.001
UACR, mg/g	9.8 (6.2–15.4)	212.4 (110.8–332.1)	< 0.001
eGFR, mL/min/1.73 m^2^	96.3 ± 14.5	52.6 ± 10.5	< 0.001

*Note:* Data are presented as mean ± standard deviation, median (interquartile range) or *n* (%), as appropriate. Normally distributed continuous variables were compared using an independent‐samples Student's *t*‐test or Welch's *t*‐test when the assumption of equal variances was not met. UACR was compared using the Mann–Whitney *U* test, and sex distribution was compared using Pearson's *χ* ^2^ test.

Abbreviations: —, not applicable; DN, diabetic nephropathy; eGFR, estimated glomerular filtration rate; HbA1c, glycated hemoglobin; UACR, urinary albumin‐to‐creatinine ratio.

The entire protocol for this human study, including experimental design, methodology, and data analysis procedures, was approved by the Henan Provincial People's Hospital Ethics Committee. All participants provided written informed consent. All experimental procedures involving human subjects were conducted in accordance with the ethical principles for medical research outlined in the Declaration of Helsinki.

### 2.2 Cell Culture and Treatment

Human podocytes (HPCs, cat. No. CP‐H075) were purchased from Procell (Wuhan, China). Cells were cultured in complete RPMI‐1640 medium (Procell) supplemented with 10% fetal bovine serum, 1% Insulin‐Transferrin‐Selenium additive, and 100 U/mL penicillin–streptomycin at 37°C under 5% CO_2_. To induce differentiation into a mature podocyte phenotype, cells were first cultured at 33°C (permissive condition) until reaching approximately 70% confluence to allow proliferation. The temperature was then shifted to 37°C (nonpermissive condition), and the cells were maintained for 10–14 days to promote differentiation. Differentiated cells were used for all experiments. Cell lines were authenticated using short tandem repeat (STR) profiling and confirmed free of mycoplasma contamination before experimentation (MycoAlert Detection Kit, Lonza Group Ltd., Basel, Switzerland).

To evaluate glucose‐specific effects, cells were treated for 48 h under the following conditions: normal glucose (NG, 5.5 mM d‐glucose), high mannitol (HM, 5.5 mM d‐glucose plus 24.5 mM d‐mannitol) as an osmotic control, and high glucose (HG, 30 mM D‐glucose). Based on existing literature [26, 27] and our preliminary findings, these concentrations were selected. To further assess the functional role of the PLAUR‐suPAR axis, cells were exposed to recombinant human suPAR protein (10 ng/mL; R&D Systems, Minneapolis, MN, USA) for 24 h. The treatment concentration and duration were selected according to previous studies [12, 28, 29] and confirmed in preliminary experiments.

### 2.3 Lentiviral Transduction and Stable Cell Line Establishment

Lentiviral vectors for overexpression and short hairpin RNA (shRNA)‐mediated knockdown targeting ELAVL1 and PLAUR genes, along with corresponding negative control empty vectors, were designed and packaged by GeneChem (Shanghai, China). Podocytes in the logarithmic growth phase were seeded at an appropriate density. When cells reached approximately 50%–60% confluence, viral transduction was performed in accordance with the manufacturer's protocol, using the predetermined optimal multiplicity of infection (MOI) for each viral preparation, supplemented with 5 μg/mL Polybrene to enhance infection efficiency. Seventy‐two hours after infection, the culture medium was replaced with complete medium supplemented with 2 μg/mL puromycin to initiate selection, which was maintained for 2–3 weeks until stable monoclonal cell lines were established. Overexpression or knockdown efficiency was validated by Western Blot. The following stable cell lines were ultimately established: ELAVL1‐overexpressing (OE‐ELAVL1), ELAVL1‐knockdown (KD‐ELAVL1), PLAUR‐overexpressing (OE‐PLAUR), PLAUR‐knockdown (KD‐PLAUR), and their respective negative controls (OE‐NC, KD‐NC).

### 2.4 Cell Counting Kit‐8 (CCK‐8)

Cell viability was evaluated with a CCK‐8 assay kit (Solarbio, Beijing, China) following the protocol provided by the supplier. Cells were plated into 96‐well culture plates at 1 × 10^4^ cells/well and allowed to adhere overnight before receiving the indicated interventions. After treatment, CCK‐8 reagent was added to each culture well at a 1:10 ratio, and the cells were further maintained at 37°C for 1–2 h. The optical density was then recorded at 450 nm with a microplate reader. The relative viability of cells was determined by applying the following formula: $\text{Viability}\;\left(\%\right)=\frac{\text{ODtreated}-\text{ODblank}}{\text{ODcontrl}-\text{ODblank}}\times 100$. Here, OD values correspond to the absorbance of treated samples, control wells, and blanks, providing a normalized measure of cell survival.

### 2.5 Flow Cytometry

Cell apoptosis was detected by flow cytometry using an Annexin V‐FITC/PI Apoptosis Detection Kit (Solarbio). All specific steps were strictly followed according to the manufacturer's instructions. Podocytes were briefly digested with trypsin, transferred to centrifuge tubes, and then resuspended in 1× binding buffer, with the cell density adjusted to 1–5 × 10^6^ cells/mL. Then, 100 μL of the prepared cell suspension was placed into a 5 mL flow cytometry tube, followed by the addition of 5 μL Annexin V‐FITC. The mixture was gently combined and kept at room temperature away from light for 5 min. Subsequently, the samples were mixed with 5 μL propidium iodide and 400 μL PBS, followed by immediate analysis using flow cytometry.

### 2.6 Terminal Deoxynucleotidyl Transferase‐Mediated dUTP Nick‐End Labeling (TUNEL)

A TUNEL assay kit (Beyotime, Shanghai, China) was used to detect apoptosis in cell slides. Cell samples were immobilized using 4% paraformaldehyde for 10 min at 37°C. Following paraffin removal, the tissue slices and prepared cell coverslips were incubated with 20 μg/mL Proteinase K. Sections were treated with DNase‐free Proteinase K at room temperature for 30 min, and then incubated with the TdT reaction solution at 37°C for 1 h under light‐protected conditions. After nuclear labeling with DAPI (4′,6‐diamidino‐2‐phenylindole), fluorescent images were obtained under a fluorescence imaging system. Quantitative image processing was subsequently performed using ImageJ software (National Institutes of Health, Bethesda, Maryland, USA). For each group, apoptotic cells were assessed by counting positively stained cells in no fewer than six randomly selected fields.

### 2.7 Cytoskeleton Staining

Cells were incubated in 4% paraformaldehyde at 37°C for 15 min for fixation, then treated with PBS supplemented with 0.3% Triton X‐100 to allow permeabilization, followed by blocking in 5% BSA for 30 min. Subsequently, cells were stained with Alexa Fluor 488 phalloidin (Thermo Fisher Scientific, Waltham, MA, USA) to visualize F‐actin for 30 min, and nuclei were stained with DAPI for 15 min. Changes in podocyte cytoskeletal structure were observed under a confocal microscope. Mean F‐actin fluorescence intensity was quantified with ImageJ software.

### 2.8 Transwell

Following trypsin digestion, the cells were collected and rinsed twice using serum‐free culture medium, then resuspended before cell numbers were determined. A total of 200 μL of cell suspension containing 1 × 10^5^ transfected cells was added to the upper chamber of a Transwell insert (Becton Dickinson, Franklin Lakes, NJ, USA). Then, the lower compartment was filled with 600 μL complete medium, and the plate was maintained at 37°C for 24 h to permit cell settling and adherence. After incubation, the insert was taken out, cells remaining on the upper membrane surface were carefully removed, and the membrane was rinsed with PBS. Migrated cells on the lower side were fixed with 4% paraformaldehyde for 30 min and stained with crystal violet for 25 min. Stained cells were observed and photographed under an optical microscope, and cell counting was performed using ImageJ software.

### 2.9 RNA Immunoprecipitation (RIP)

The RIP assay was performed using the Magna RIP RNA‐Binding Protein Immunoprecipitation Kit (Millipore, Bedford, MA, USA) according to the manufacturer's instructions. Cells were collected and disrupted in complete RIP lysis buffer. The resulting lysates were then incubated at 4°C overnight with beads conjugated to anti‐ELAVL1 antibody (ab200342; Abcam, Cambridge, MA, USA) or control IgG. RNA was then extracted and the enrichment level of target RNA was identified by RT‐qPCR.

### 2.10 mRNA Stability Assay

Transfected cells were cultured for 24 h and then treated with 4 μM actinomycin D (Cat. No. HY‐17559, MedChemExpress [MCE], Monmouth, NJ, USA) to inhibit transcription. Cells were collected at 0, 3, 6, and 9 h posttreatment. RNA was extracted, and PLAUR mRNA levels were detected by RT‐qPCR.

### 2.11 Experimental Animals

Six‐week‐old male C57BLKS/J‐LepRdb/LepRdb (db/db) mice and their littermate C57BLKS/J‐LepRdb/+ (db/m) controls were purchased from Cavens Laboratory Animal Company (Jiangsu, China). All mice were housed in a specific pathogen‐free facility under a standard 12‐h light/dark cycle. Room temperature was maintained at approximately 24°C ± 2°C with a humidity of about 40%. Animals were fed a standard diet with free access to water. All mice underwent a one‐week acclimatization period. Validation of the DN model required non‐fasting blood glucose measurements in db/db mice to exceed 13.9 mmol/L.

### 2.12 Animal Experimental Design

To investigate the functional role of the ELAVL1–PLAUR signaling axis in DN, 30 db/db mice were randomly assigned into three experimental cohorts, each containing 10 animals. Adeno‐associated virus (AAV)‐shControl group: received injection of AAV carrying a scrambled control sequence; AAV‐shElavl1 group: received injection of AAV carrying shRNA targeting the Elavl1 gene for in vivo knockdown of ELAVL1 expression; AAV‐shPlaur group: received injection of AAV carrying shRNA targeting the Plaur gene for in vivo knockdown of PLAUR expression. The normal control group consisted of Db/m mice.

To verify the functional relationship of the ELAVL1‐PLAUR‐suPAR axis in DN, a rescue experiment was designed. Thirty male db/db mice aged 6 weeks were randomly assigned to three experimental groups, with 10 mice included in each group: AAV‐shElavl1 group; AAV‐shElavl1 + oePlaur group: co‐injected with AAV‐shElavl1 and AAV carrying a Plaur overexpression vector; AAV‐shElavl1 + suPAR group: received injection of AAV‐shElavl1 followed by intraperitoneal injection of recombinant mouse suPAR protein.

### 2.13 Animal Treatments

AAV Injection: Validated shRNA or overexpression sequences were packaged into AAV9 using the nphs1 (a podocyte‐specific marker) promoter, produced and purified by HanBio (Shanghai, China). The viral stock was diluted in sterile, endotoxin‐free physiological saline to the required volume (100 μL) at a dose of 1 × 10^12^ vector genomes (vg) per mouse. For injection, mice were placed in a restrainer, and their tails were immersed in warm water (approx. 40°C) for 30–60 s to dilate the veins. Using an insulin syringe, the virus solution was slowly and steadily injected into a lateral tail vein. All AAV injections were performed when mice were 7 weeks old (a time when db/db mice exhibit stable hyperglycemia and obesity, but significant glomerular injury has not yet commenced). At Week 4 after administration, kidney tissues from one to two randomly chosen mice in each group were collected to verify the efficiency of gene silencing or overexpression. The db/m control mice received the same volume of normal saline.

Recombinant mouse suPAR protein (Cat. No. HY‐P77276) was purchased from MCE. Four weeks following AAV administration, mice assigned to the AAV‐shElavl1 + suPAR group were treated with suPAR protein. Mice received intraperitoneal injections of the protein at 20 μg per animal each time, administered three times weekly until the study endpoint at 22 weeks of age. The db/db + shElavl1 mice were administered the same volume of PBS by intraperitoneal injection as the vehicle control. The administration protocol for suPAR was referenced from prior studies [28, 30] and refined in pilot experiments.

### 2.14 Biochemical Indicators Measurement

Biomarker monitoring included body weight measurement every 2 weeks and assessment of fasting blood glucose (after a 6‐h fast via tail vein sampling) every 2 weeks, with detailed records kept. Before sample collection, mice were placed in metabolic cages for 24‐h urine collection. An automated biochemical analyzer (Beckman Coulter Inc. Miami, FL, USA) was used to measure urinary creatinine (UCr) and 24‐h urinary protein (24‐h UP) levels. UACR was determined to assess the degree of proteinuria. At 22 weeks of age, all mice were placed under anesthesia with 1% sodium pentobarbital before sample collection. Blood was collected via cardiac puncture and allowed to clot for 2 h. Serum was separated by centrifugation at 14 000 rpm for 20 min at 4°C. Scr concentrations in serum were measured following the manufacturer's protocol using a commercial kit (Cat. No. C011‐2, Jiancheng Bioengineering Institute, Nanjing, China).

### 2.15 Tissue Sample Collection

Mice were then euthanized, and both kidneys were rapidly excised and weighed. A portion of renal tissue was fixed in 4% paraformaldehyde for subsequent histological examination. The remaining renal cortex was quickly separated, snap‐frozen in liquid nitrogen, and stored at −80°C.

### 2.16 Renal Histopathological Analysis

Renal specimens from mice were immersed in 4% paraformaldehyde for 24 h, followed by routine paraffin embedding. The paraffin blocks were then cut into 4‐μm slices with a microtome and placed onto glass slides. Routine staining was performed using a Periodic Acid‐Schiff (PAS) kit (Cat. No. C0142S, Beyotime, China) and a Masson's trichrome staining kit (Cat. No. G1340, Solarbio, China) according to the manufacturers' instructions. The stained tissue sections were examined using a light microscope. PAS staining was used to assess the degree of mesangial matrix expansion, while Masson's staining highlighted renal interstitial fibrosis. Representative images were captured. The PAS‐positive and Masson‐positive areas within the glomerular tuft area were quantified using ImageJ software. The glomerular area was estimated based on the average area of at least 20 randomly selected glomeruli per group.

### 2.17 Immunohistochemical Staining

Tissue sections were first deparaffinized in xylene twice for 10 min each at room temperature, followed by rehydration. Endogenous peroxidase activity was blocked by incubation with 3% H_2_O_2_ for 10 min. Antigen retrieval was performed by heating the sections in citrate buffer at 98°C for 20 min. Primary antibody staining was then performed at room temperature for 90 min, after which the slides were exposed to a biotin‐conjugated secondary antibody for 30 min at 37°C. DAB chromogen was applied to develop the immunoreactive signal for 10–20 min, after which the slides were lightly counterstained with hematoxylin for 2 min to visualize cell nuclei. For negative controls, the sections were treated with phosphate‐buffered saline (PBS) in the absence of the primary antibody to assess background staining. Details regarding the primary antibodies are summarized in Table S2. Representative micrographs were obtained, and ImageJ was used to calculate the proportion of immunopositive regions within each glomerulus.

### 2.18 Immunofluorescence

Podocytes were cultured in six‐well plates according to the experimental grouping, followed by immobilization using 4% paraformaldehyde. Cells were first treated with 0.1% Triton X‐100 to allow membrane penetration, followed by incubation in 5% BSA for 30 min at room temperature to minimize nonspecific staining. Subsequently, cells were incubated with primary antibodies at 4°C overnight. Cells were then incubated with fluorescently labeled secondary antibodies at 37°C in the dark for 1–2 h. Nuclei were then labeled with DAPI for 10 min. Fluorescent signals were captured under a fluorescence microscope, followed by ImageJ‐based quantification.

Paraffin sections of human renal biopsies and mouse kidneys were deparaffinized in xylene. Endogenous peroxidase activity was quenched using methanol‐prepared ethanol and 2% hydrogen peroxide solution. Antigen retrieval was performed by heating the sections in 0.1 mol/L sodium citrate buffer at pH 6.0 and 95°C for 15 min. After blocking with 5% normal goat serum for 1 h, sections were incubated with primary antibodies at 4°C overnight. This was followed by incubation with fluorescently labeled secondary antibodies at room temperature for 1 h. For nuclear staining, sections were counterstained with a solution containing DAPI. Imaging was performed using a fluorescence microscope, and images were analyzed with ImageJ software. Information on primary antibodies used is provided in Table S2.

### 2.19 Enzyme‐Linked Immunosorbent Assay (ELISA)

The levels of suPAR in cell culture supernatants or serum were measured using commercially available Human suPAR ELISA Kit (Cat. No. E‐EL‐H2584, Elabscience, Wuhan, China) or Mouse suPAR ELISA Kit (Cat. No. BY‐EM221788, Byabscience, Nanjing, China), respectively. All experimental steps were performed following the instructions provided by the corresponding reagent or kit suppliers.

Inflammatory cytokine levels were assessed by measuring TNF‐α and IL‐6 concentrations using Mouse/Human TNF‐α and IL‐6 ELISA kits from Beyotime, respectively. All procedures followed the kit instructions. Briefly, samples, biotin‐labeled antibodies, and horseradish peroxidase (HRP)‐conjugated reagents were added sequentially according to the protocol. After tetramethylbenzidine (TMB) substrate development, absorbance was read at 450 nm using a microplate reader. Concentrations were calculated based on the standard curve.

### 2.20 Western Blot

Total protein was extracted from tissues and cells using radio‐immunoprecipitation assay (RIPA) buffer (Beyotime) according to the kit instructions. Protein concentration was determined using a bicinchoninic acid (BCA) Protein Assay Kit (Beyotime). According to the results obtained from protein concentration determination, identical quantities of protein samples were prepared and resolved on 10% SDS–polyacrylamide gels by electrophoresis, which was carried out for nearly 2 h. Proteins were then transferred to a membrane. The membrane was incubated in 5% skim milk for 2 h at room temperature to block nonspecific binding, and was then exposed to the primary antibodies overnight at 4°C. Following incubation with the primary antibody, the membrane was treated with the corresponding secondary antibody at 37°C for 2 h. Following washing steps, protein bands were visualized using a fluorescence reagent. Protein expression levels were quantified using ImageJ software. Antibody information is detailed in Table S2.

### 2.21 Total RNA Preparation and Real‐Time Quantitative PCR Analysis

Total cellular RNA was extracted using TRIzol reagent purchased from Thermo Fisher Scientific. Complementary DNA (cDNA) was synthesized using PrimeScript RT Master Mix (Takara, Shiga, Japan). Quantitative PCR (qPCR) was performed using SYBR Premix Ex Taq II (Takara, Shiga, Japan) on a 7500 Real‐Time PCR System (Applied Biosystems, USA). Gene expression levels were normalized to β‐actin, and relative transcript abundance was calculated using the 2^‐ΔΔCt^ approach. Details of the primer sequences are listed in Table S3.

### 2.22 Statistical Analysis

Statistical analysis was performed using GraphPad Prism version 10.0 (GraphPad Software, CA, USA). Data are presented as mean ± standard deviation (SD). Normality and homogeneity of variance were assessed before group comparisons. Differences in baseline clinical characteristics between DN patients and healthy controls were analyzed using an independent Student's *t*‐test or Mann–Whitney *U* test for continuous variables and the chi‐square test or Fisher's exact test for categorical variables. When three or more groups were compared, data were analyzed using one‐way ANOVA, with either Tukey or Bonferroni multiple‐comparison correction applied where appropriate. Differences between two groups were evaluated using a two‐sided independent Student's *t*‐test. Correlations between ELAVL1/PLAUR expression, serum suPAR levels, UACR, and eGFR were assessed using Pearson or Spearman correlation analysis according to data distribution. A *p* value below 0.05 was considered statistically significant. All assays were performed in no fewer than three independent runs, unless stated otherwise, and yielded reproducible findings.

## 3 Results

### 3.1 The ELAVL1‐PLAUR‐suPAR Axis Contributes to the Pathogenesis of DN

**FIGURE 1:**
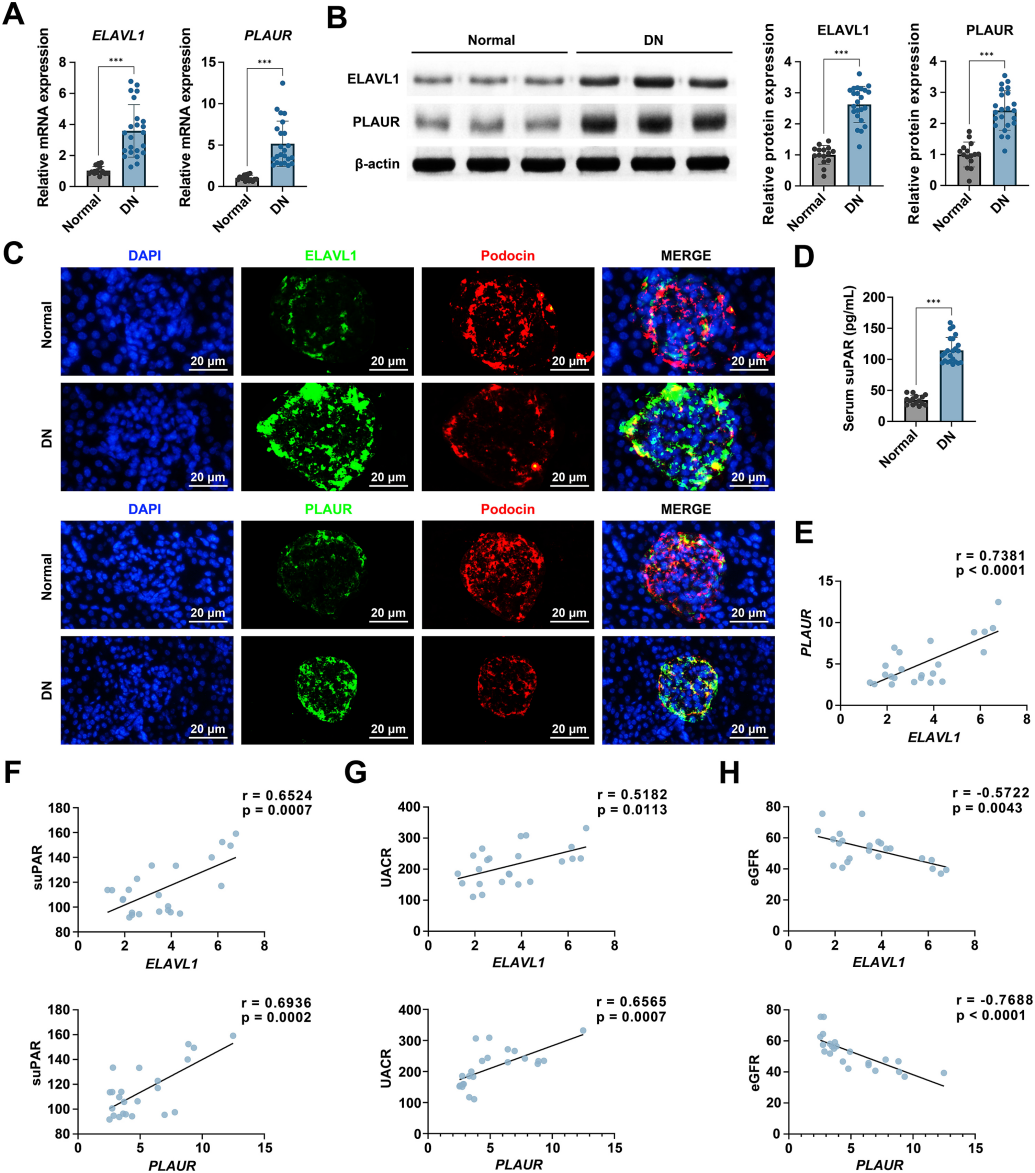
The ELAVL1‐PLAUR‐suPAR Axis Contributes to the Pathogenesis of DN. (A, B) RT‐qPCR and Western blot analysis showing significantly increased mRNA (A) and protein (B) levels of ELAVL1 and PLAUR in renal tissues of DN patients compared to normal controls (Normal) (DN group, *n* = 23; Normal group, *n* = 15). (C) Immunofluorescence double staining shows colocalization of ELAVL1 or PLAUR (green) with the podocyte marker Podocin (red) in glomeruli. Nuclei were counterstained with DAPI (blue). (D) ELISA revealing significantly upregulation of serum suPAR levels in DN patients compared to healthy controls (HC) (DN group, *n* = 23; HC group, *n* = 23). (E, F) Positive correlation between ELAVL1 and PLAUR mRNA expression in renal tissues (E), and both positively correlated with serum suPAR levels (F) (*n* = 23). (G, H) The level of ELAVL1 and PLAUR in glomerular podocytes positively correlated with the urinary albumin‐to‐creatinine ratio (UACR) (G) and negatively correlated with the estimated glomerular filtration rate (eGFR) (H) (*n* = 23). Data are presented as mean ± SD. ns, not significant; ***p < 0.001.

Analysis of clinical renal biopsy samples revealed that both mRNA and protein expression levels of ELAVL1 and PLAUR were significantly elevated in the renal tissues of DN patients compared to normal controls (Figure 1A,B). Immunofluorescence double staining further confirmed that ELAVL1 and PLAUR protein expression was enriched in response to podocyte injury (Figure 1C). ELISA showed that serum suPAR levels were markedly higher in DN patients than in healthy controls (Figure 1D). Correlation analysis demonstrated a positive relationship between ELAVL1 and PLAUR levels in renal tissues (Figure 1E), and both were significantly positively correlated with serum suPAR concentrations (Figure 1F), suggesting a potential intrinsic link between ELAVL1 upregulation and increased PLAUR expression and circulating suPAR. Notably, expression levels of ELAVL1 and PLAUR correlated positively with the UACR (Figure 1G) and inversely with the eGFR (Figure 1H). These findings indicate that dysregulation of the ELAVL1‐PLAUR‐suPAR axis is closely associated with podocyte injury in DN.

### 3.2 ELAVL1 Promotes suPAR Release in Podocytes by Directly Binding to and Stabilizing PLAUR mRNA

**FIGURE 2:**
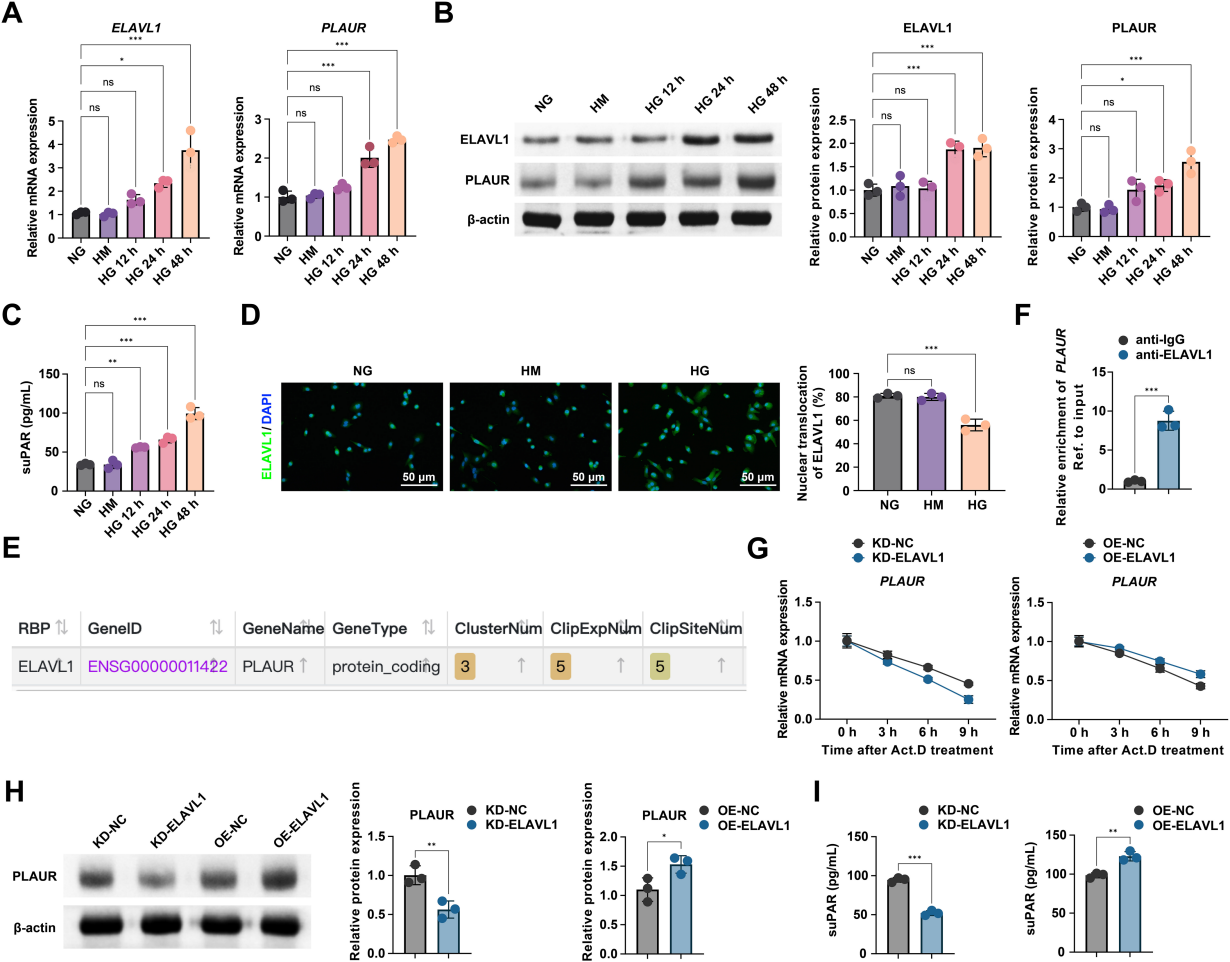
ELAVL1 Promotes suPAR Release in Podocytes by Directly Binding to and Stabilizing PLAUR mRNA. (A, B) Time‐dependent upregulation of ELAVL1 and PLAUR mRNA (A) and protein (B) expression in human podocytes treated with HG, as detected by RT‐qPCR and Western blot, respectively. (C) Time‐dependent upregulation of cellular supernatant suPAR concentration detected by ELISA. (D) Immunofluorescence detection of ELAVL1 (green) in HG‐treated podocytes. Nuclei were stained with DAPI (blue). Nuclear translocation of ELAVL1 was quantified by calculating the ratio of nuclear staining intensity to total staining intensity (*n* = 3 independent experiments). (E) ENCORI database prediction of binding sites between ELAVL1 and PLAUR mRNA. (F) RIP assay confirms the binding of ELAVL1 protein to PLAUR mRNA. (G) Actinomycin D assay shows that knockdown or overexpression of ELAVL1 shortened or prolonged the half‐life of PLAUR mRNA, respectively. (H, I) Silencing or overexpression of ELAVL1 correspondingly decreased or increased PLAUR protein levels (H) and supernatant suPAR concentration (I), detected by Western blot and ELISA, respectively. Data are presented as mean ± SD (*n* = 3 independent experiments). ns, not significant; *p < 0.05, **p < 0.01, ***p < 0.001.

In vitro experiments using HPCs treated with HG showed time‐dependent significant upregulation of ELAVL1 and PLAUR expression, as well as increased suPAR concentration in the cell supernatant (Figure 2A–C), recapitulating the clinical observations. Notably, immunofluorescence analysis revealed nucleo‐cytoplasmic shuttling of ELAVL1 upon HG treatment, with ELAVL1 antibody signal shifting from the nucleus to the cytoplasm (Figure 2D). These data collectively suggest that HG may induce translocation and activation of ELAVL1, enabling it to participate in PLAUR expression regulation by modulating PLAUR mRNA stability, thereby promoting suPAR release. At the mechanistic level, bioinformatic analysis via the ENCORI database predicted a potential direct interaction between ELAVL1 and PLAUR mRNA (Figure 2E). RIP assay directly confirmed the binding of ELAVL1 protein to PLAUR mRNA (Figure 2F). Functional studies demonstrated that under HG conditions, ELAVL1 knockdown significantly shortened the half‐life of PLAUR mRNA, whereas ELAVL1 overexpression extended it (Figure 2G). Consistent with this, ELAVL1 silencing reduced both PLAUR protein levels and suPAR concentration in the supernatant, while its overexpression had the opposite effect (Figure 2H,I). These results demonstrate that ELAVL1 promotes suPAR release from podocytes by post‐transcriptionally upregulating PLAUR expression through direct mRNA binding and stabilization.

### 3.3 Knockdown of ELAVL1 or PLAUR Attenuates HG‐Induced Podocyte Injury, Inflammation, and Dysfunction

**FIGURE 3:**
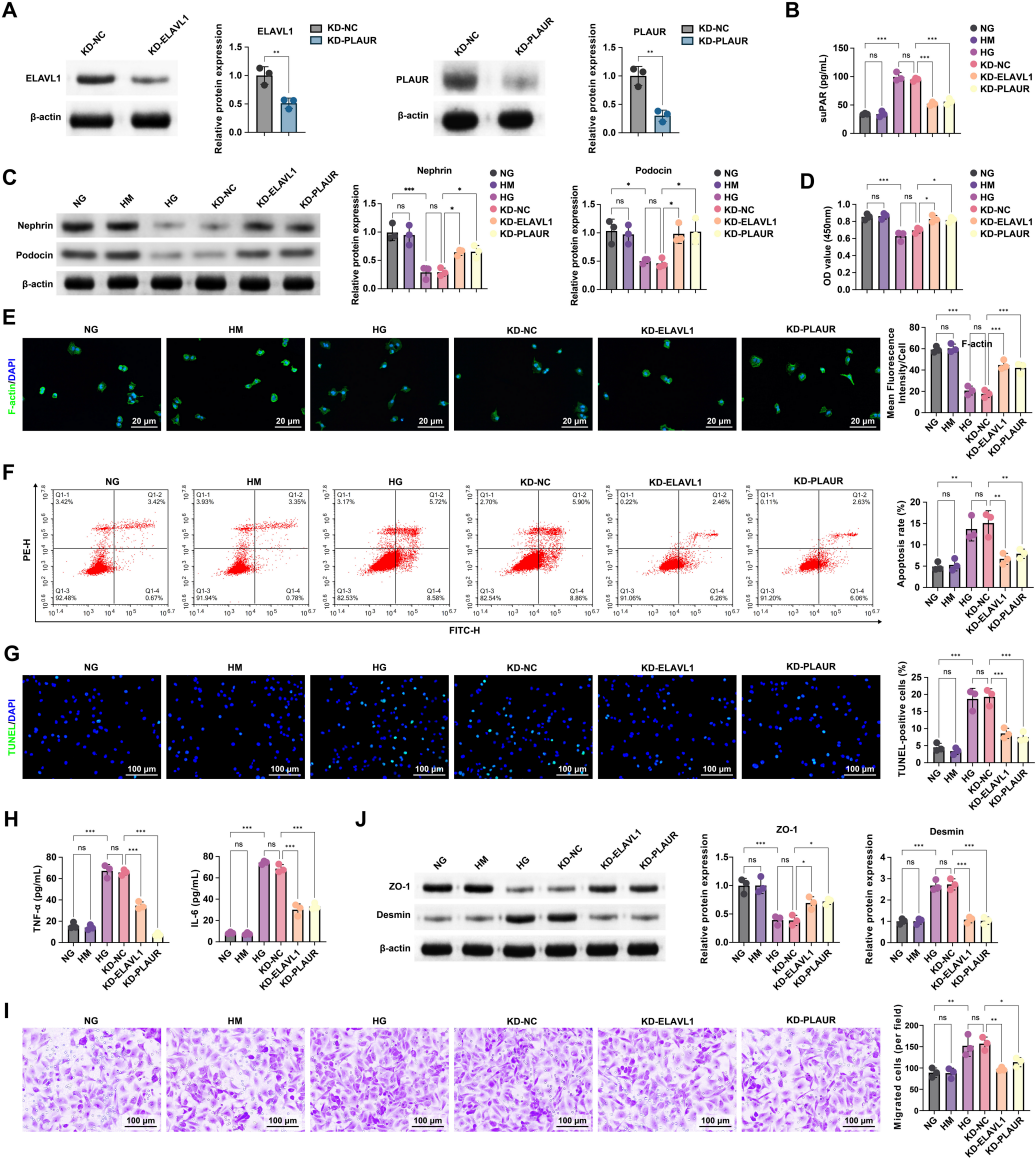
Knockdown of ELAVL1 or PLAUR attenuates HG‐induced podocyte injury, inflammation, and dysfunction. (A) Western Blot validation of ELAVL1 or PLAUR knockdown efficiency in podocytes. Lentivirus‐transfected cells were stimulated with HG (30 mM) for 48 h. Untransfected cells treated with normal glucose (NG, 5.5 mM), mannitol (HM, 24.5 mM mannitol + 5.5 mM glucose), or HG for 48 h served as controls. (B) suPAR concentration in cell culture supernatant measured by ELISA. (C) Protein expression levels of Nephrin and Podocin detected by Western blot. (D) Cell viability assessed by CCK‐8 assay. (E) Representative immunofluorescence images and quantitative analysis of F‐actin in podocytes. (F, G) Apoptosis detected by flow cytometry (F) and TUNEL assay (G). (H) Pro‐inflammatory cytokines TNF‐α and IL‐6 levels in cell culture supernatant measured by ELISA. (I) Cell migration ability assessed by Transwell assay. (J) Protein expression levels of ZO‐1 and Desmin detected by Western blot. Data are presented as mean ± SD (*n* = 3 independent experiments). ns, not significant; *p < 0.05, **p < 0.01, ***p < 0.001.

To investigate the functional roles of ELAVL1 and PLAUR in DN, we first established stable knockdown models in podocytes. Knockdown efficiency was confirmed by Western Blot (Figure 3A). Both knockdowns effectively reduced the HG‐induced increase in suPAR concentration in the cell supernatant (Figure 3B).

Because disruption of slit diaphragm integrity is a hallmark of podocyte injury in DN, we next evaluated the expression of podocyte‐specific structural proteins. Western blot analysis demonstrated that HG markedly decreased the expression of Nephrin and Podocin compared with the NG group, whereas knockdown of either ELAVL1 or PLAUR significantly restored the expression of both proteins (Figure 3C), indicating preservation of podocyte structural integrity.

Consistent with these findings, ELAVL1 or PLAUR knockdown markedly alleviated HG‐induced podocyte injury. CCK‐8 assays showed improved cell viability (Figure 3D), while immunofluorescence staining revealed restoration of the F‐actin cytoskeletal architecture (Figure 3E). Flow cytometry (Figure 3F) and TUNEL staining (Figure 3G) further demonstrated significantly reduced apoptotic cell death. Moreover, ELISA showed significantly decreased secretion of the pro‐inflammatory cytokines TNF‐α and IL‐6 following ELAVL1 or PLAUR knockdown (Figure 3H).

Beyond attenuating podocyte injury, ELAVL1 or PLAUR silencing also improved podocyte function. Transwell assays demonstrated reduced aberrant migratory activity (Figure 3I), while western blot analysis revealed restoration of the epithelial marker ZO‐1 accompanied by decreased Desmin expression (Figure 3J), indicating attenuation of HG‐induced podocyte dedifferentiation. Collectively, these findings demonstrate that inhibition of the ELAVL1–PLAUR axis not only suppresses apoptosis and inflammation but also preserves slit diaphragm integrity, cytoskeletal organization, and podocyte phenotype under diabetic conditions.

### 3.4 PLAUR‐suPAR Mediates the Protective Effects of ELAVL1 Knockdown in HG‐Induced Podocytes

**FIGURE 4:**
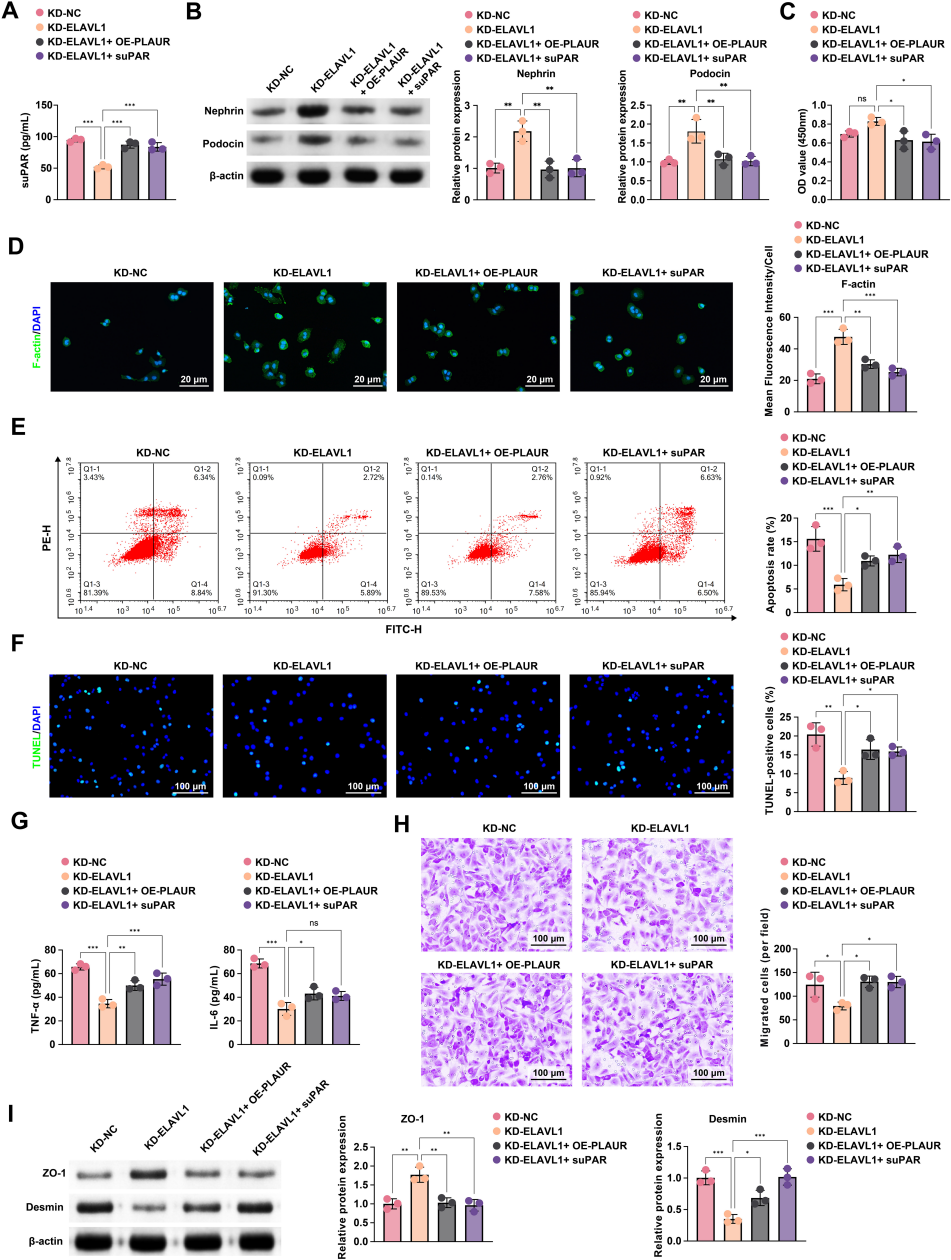
PLAUR‐suPAR Mediates the Protective Effects of ELAVL1 Knockdown in HG‐Induced Podocytes. (A) suPAR concentration in cell culture supernatant measured by ELISA. (B) Protein expression levels of Nephrin and Podocin detected by Western blot. (C) Cell viability assessed by CCK‐8 assay. (D) Representative immunofluorescence images and quantitative analysis of F‐actin in podocytes. (E, F) Apoptosis detected by flow cytometry (E) and TUNEL assay (F). (G) Pro‐inflammatory cytokines TNF‐α and IL‐6 levels in cell culture supernatant measured by ELISA. (H) Cell migration ability assessed by Transwell assay. (I) Protein expression levels of ZO‐1 and Desmin detected by Western blot. Data are presented as mean ± SD (*n* = 3 independent experiments). ns, not significant; *p < 0.05, **p < 0.01, ***p < 0.001.

To verify whether ELAVL1 function depends on the PLAUR‐suPAR axis, rescue experiments were performed using PLAUR overexpression or exogenous suPAR supplementation. Both interventions effectively restored the suPAR release that had been suppressed by ELAVL1 knockdown, returning its supernatant concentration to levels comparable to the HG control (Figure 4A). Moreover, in podocytes with confirmed ELAVL1 knockdown, these treatments partially reversed the protective effects resulting from ELAVL1 loss. Specifically, podocyte injury phenotypes that were ameliorated by ELAVL1 knockdown became re‐aggravated following rescue, with several metrics reverting to levels similar to those in the HG injury group (Figure 4B–I). These results indicate that the PLAUR‐suPAR axis functions downstream of ELAVL1 and is an essential effector required for ELAVL1‐mediated podocyte injury.

### 3.5 Podocyte‐Specific Knockdown of Elavl1 or Plaur Alleviates DN Progression in Db/Db Mice

**FIGURE 5:**
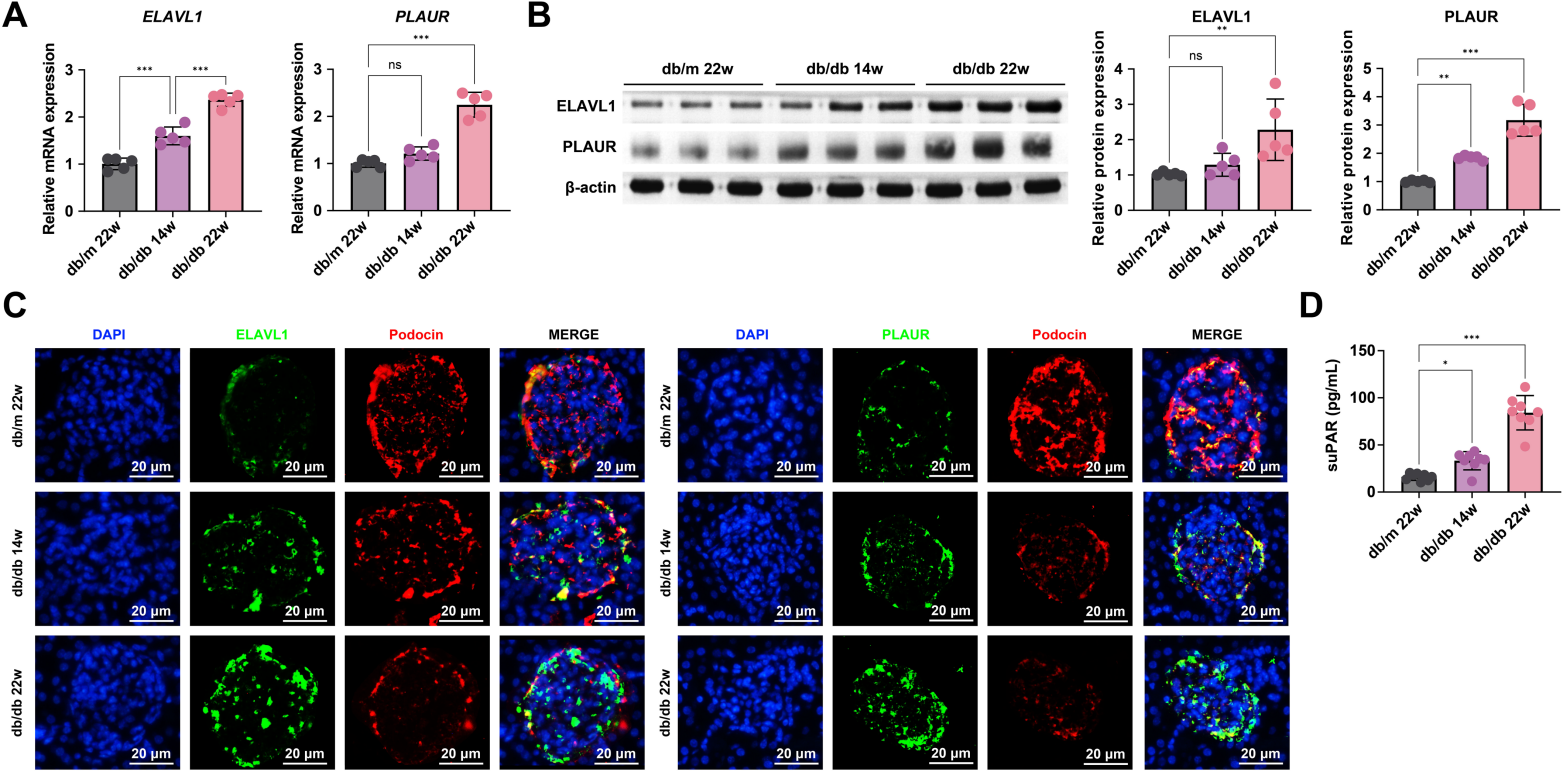
Renal Elavl1/Plaur expression and serum suPAR are upregulated with disease progression in db/db mice. (A) RT‐qPCR analysis of Elavl1 and Plaur mRNA levels in mouse kidneys. (B) Western blot analysis of Elavl1 and Plaur protein levels in mouse kidneys. (C) Immunofluorescence double staining shows co‐localization of Elavl1/Plaur (red) with the podocyte marker Podocin (green) in glomeruli of mouse kidneys. Nuclei were counterstained with DAPI (blue). (D) Serum suPAR concentration measured by ELISA. Data are presented as mean ± SD (*n* = 6 mice/group). ns, not significant; *p < 0.05, **p < 0.01, ***p < 0.001.

**FIGURE 6:**
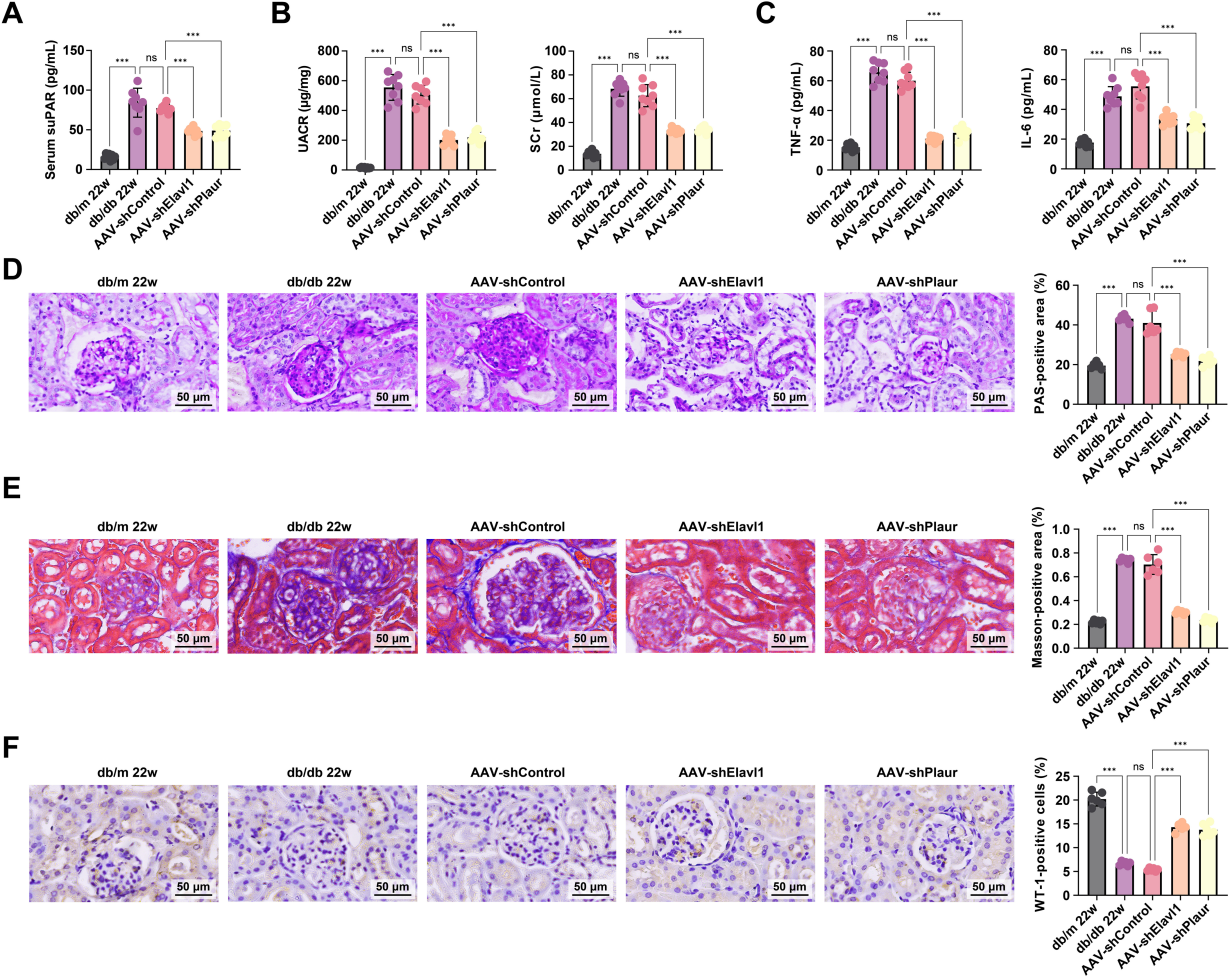
Podocyte‐specific knockdown of Elavl1 or Plaur alleviates nephropathy progression in db/db mice. (A) Serum suPAR levels. (B) UACR and Scr levels. (C) Serum levels of pro‐inflammatory cytokines TNF‐α and IL‐6. (D, E) Representative images of renal tissue PAS staining (D) and Masson staining (E), with quantitative analysis of glomerular mesangial expansion and interstitial fibrosis. (F) Representative images and quantitative analysis of immunohistochemical staining for the podocyte marker WT‐1. Data are presented as mean ± SD (*n* = 6 mice/group). ns, not significant; ***p < 0.001.

Animal experimental results indicated that during DN progression, the expression of ELAVL1 and PLAUR showed an upward trend. As the disease progressed, the expression levels of Elavl1 and Plaur in kidneys of db/db mice gradually increased, becoming significantly higher than in db/m controls by 22 weeks of age (Figure 5A–C). Concurrently, serum circulating suPAR levels showed a consistent increasing trend (Figure 5D). To clarify the in vivo function of ELAVL1 and PLAUR, we employed an AAV9‐nphs1 system to achieve podocyte‐specific knockdown of Elavl1 or Plaur in db/db mice. After treatment, silencing of either target markedly lowered circulating suPAR levels (Figure 6A), ameliorated renal function indices, including UACR and serum creatinine (Figure 6B), and reduced serum TNF‐α and IL‐6 concentrations (Figure 6C). Histopathological analysis further showed significant alleviation of glomerular mesangial matrix expansion (PAS staining) and fibrosis (Masson's trichrome staining) in the knockdown groups (Figure 6D,E). Additionally, podocyte quantification via Wilms' Tumor 1 (WT‐1) immunohistochemistry staining revealed a marked reduction in podocyte number per glomerulus in db/db mice, which was effectively ameliorated by Elavl1 or Plaur knockdown (Figure 6F).

### 3.6 PLAUR‐suPAR Mediates the Pathogenic Role of ELAVL1 In Vivo

**FIGURE 7:**
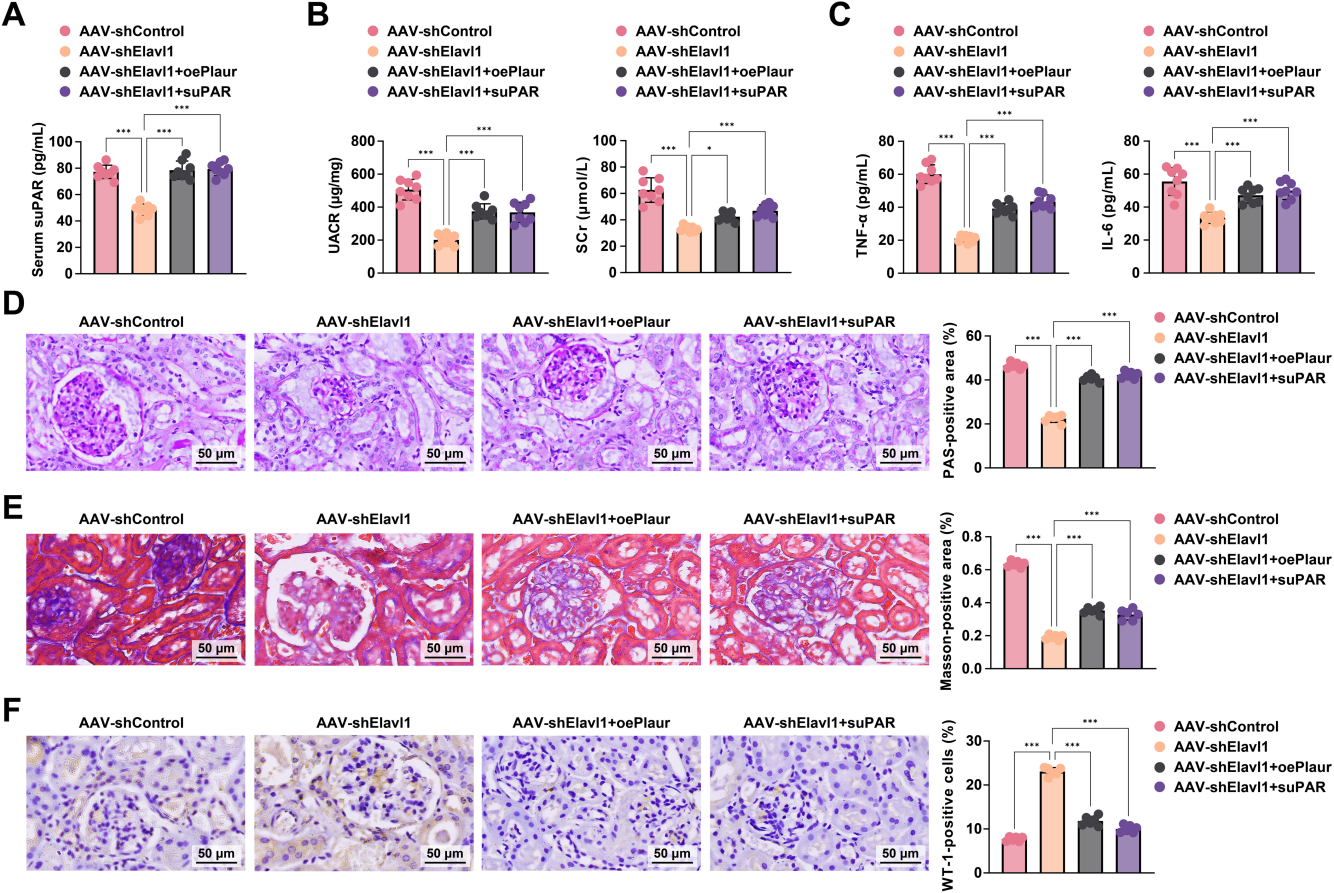
The PLAUR‐suPAR axis mediates the pathogenic role of ELAVL1 in vivo. Restoration of PLAUR expression or exogenous supplementation of suPAR protein in db/db mice with podocyte‐specific Elavl1 knockdown. (A) Serum suPAR levels. (B) UACR and Scr levels. (C) Serum levels of pro‐inflammatory cytokines TNF‐α and IL‐6. (D, E) Representative images of renal tissue PAS staining (D) and Masson staining (E), with quantitative analysis of glomerular mesangial expansion and interstitial fibrosis. (F) Representative images and quantitative analysis of immunohistochemical staining for the podocyte marker WT‐1. Data are presented as mean ± SD (*n* = 6 mice/group). ns, not significant; *p < 0.05, ***p < 0.001.

To verify the causal relationship of ELAVL1 acting through the PLAUR‐suPAR axis in vivo, rescue experiments were performed in db/db mice with podocyte‐specific Elavl1 knockdown. Forced restoration of podocyte PLAUR expression via AAV or exogenous supplementation of suPAR protein successfully elevated serum suPAR concentration back to high levels (Figure 7A). Both interventions partially reversed the renal protective effects conferred by Elavl1 knockdown: the UACR, serum creatinine, and pro‐inflammatory cytokine levels increased again (Figure 7B,C), and renal pathological damage was re‐aggravated (Figure 7D–F). These results demonstrate that ELAVL1 primarily drives the initiation and progression of DN by activating the podocyte PLAUR‐suPAR axis.

## 4 Discussion

This work combined patient‐derived specimens, in vitro experimental systems, and gene‐modified animal models to comprehensively demonstrate that the RNA‐binding protein ELAVL1 contributes importantly to podocyte damage in DN and to clarify the related downstream regulatory mechanisms. Our core findings are that under diabetic hyperglycemic conditions, ELAVL1 expression is upregulated in podocytes. The upregulated ELAVL1 directly binds to and stabilizes PLAUR mRNA, thereby increasing the expression of its encoded protein, uPAR, and the secretion of its soluble form, suPAR. The released suPAR subsequently exacerbates podocyte injury, inflammatory responses, and dysfunction in an autocrine or paracrine manner, ultimately driving the progression of DN.

As a central mechanism in DN, recent research has increasingly emphasized the role of podocyte injury in the pathogenesis of DN [31]. Various factors in the diabetic milieu can damage podocytes, leading to foot process effacement, reduced podocyte density, and even podocyte detachment or death [32]. Hyperglycemia, in particular, is a key determinant of podocyte injury in DN. Over the past decades, extensive research on podocytes under high‐glucose conditions has confirmed that hyperglycemia can alter podocyte phenotype by inducing nephrin loss, changes in extracellular matrix production/degradation, enhanced signaling of the pro‐fibrotic cytokine TGF‐β1, actin cytoskeleton remodeling, downregulation of α3β1 integrin, and podocyte hypertrophy and apoptosis [33, 34].

The primary significance of this study lies in establishing ELAVL1 as a pivotal regulatory node linking metabolic stress to podocyte injury. Previous DN‐related clinical studies have reported that histological ELAVL1 expression is an independent risk factor for kidney progression 6 months postrenal biopsy in DN patients [35]. More recent research further indicates that ELAVL1 expression is significantly upregulated in DN patients and may serve as a potential biomarker for DN identification and monitoring [36]. Consistent with these findings, our work demonstrates a direct correlation between upregulated ELAVL1 expression and the severity of proteinuria as well as the decline in renal function in DN patients. This suggests that ELAVL1 upregulation is not merely a concomitant phenomenon of DN but likely a key event actively involved in disease pathogenesis. Our subsequent work extended the focus to podocytes, confirming through in vitro and in vivo models that intervention targeting ELAVL1 effectively mitigates podocyte injury, inflammation, and functional abnormalities, thereby significantly ameliorating DN progression. This provides direct functional evidence supporting ELAVL1 as a therapeutic target for DN and further corroborates previous similar research findings.

On a mechanistic level, we have elucidated the downstream pathway through which ELAVL1 exerts its effects, namely the ELAVL1‐PLAUR‐suPAR axis. uPAR is a highly glycosylated, lipid‐anchored receptor present on the surface of various cells, involved in cell adhesion, migration, invasion, and tissue remodeling. suPAR is its soluble, biologically active form, produced by cleavage via GPI‐specific phospholipase C or cathepsin G. suPAR is an innate immune system‐derived risk factor for acute and chronic kidney diseases [37], and its levels are a strong predictor of progressive renal function decline [38, 39, 40]. As oxidative stress and inflammation (involving multiple immune cell subtypes) are key components of DN pathogenesis [41, 42], the inflammatory and immune‐related molecule suPAR is under exploration as a potential novel biomarker for DN. Clinical studies confirm that serum suPAR levels are elevated in DN patients and demonstrate strong associations with progressive renal dysfunction and marked histopathological damage [14]. The pleiotropic functions of suPAR include inhibiting neutrophil exocytosis, binding to uPA and vitronectin, promoting angiogenesis and chemotaxis, and critically, engaging β3 integrin to induce glomerular podocyte injury [43]. Despite these insights, the precise upstream regulatory mechanisms responsible for dysregulated suPAR generation in DN are still largely undefined. Our work fills this gap by revealing that hyperglycemia, via ELAVL1, “amplifies” PLAUR expression at the post‐transcriptional level, representing a key mechanism driving excessive suPAR release. This may offer a fresh viewpoint for interpreting where circulating suPAR originates in DN, while also linking, in a biologically coherent way, two molecules that have previously been implicated in kidney disease: ELAVL1, an RNA‐binding protein regulating mRNA stability, and suPAR, a circulating pathogenic factor, with PLAUR acting as the pivotal molecular intermediary. Furthermore, the rescue experiments performed both in vitro and in vivo establish PLAUR and suPAR as critical downstream effectors of ELAVL1, providing compelling evidence for the causality of this regulatory axis. These findings validate the sequential “ELAVL1‐PLAUR‐suPAR” signaling axis, establishing it as a central mechanism through which ELAVL1 mediates its pathogenic activity. Moreover, this pathway appears to play a predominant role in driving the observed disease‐related effects attributed to ELAVL1. Consequently, therapeutic targeting of this axis represents a promising strategy for DN intervention.

This study has certain limitations. First, although we confirmed ELAVL1's regulation of PLAUR, the specific upstream signaling pathways through which hyperglycemia induces ELAVL1 upregulation (e.g., which kinases mediate its nucleocytoplasmic shuttling or protein stability changes) are not fully elucidated. Second, suPAR‐induced podocyte injury involves a complex downstream signaling network. This study lacks a more comprehensive analysis of the associated pathways, and the precise spatiotemporal activation profile and crosstalk with other injury pathways (e.g., TGF‐β, Notch) warrant further exploration. Finally, this study primarily relies on the db/db mouse model. While this model simulates Type 2 diabetic metabolic syndrome, its nephropathy phenotype often does not progress to advanced nodular sclerosis. Future validation of the long‐term efficacy of therapeutic strategies is needed in more severe composite models or organoid models.

## 5 Conclusion

This study identifies a novel pathogenic pathway in DN wherein hyperglycemia‐induced ELAVL1 upregulates PLAUR expression and promotes suPAR release from podocytes, leading to renal inflammation and injury. This discovery not only deepens the understanding of the molecular mechanisms underlying podocyte injury in DN but also lays a solid theoretical foundation for developing novel diagnostic biomarkers and therapeutic methods targeting key molecules in this axis. Future studies may investigate the therapeutic potential of small‐molecule inhibitors or neutralizing antibodies directed against this signaling pathway, thereby offering novel strategies for the prevention and clinical management of DN.

## Author Contributions

**DanDan Xu:** conceptualization, methodology, validation, data curation, writing – riginal draft preparation, writing – review and editing, supervision, project administration. **Liang Xu:** methodology, software, validation, formal analysis, investigation, visualization. **LinLin Li:** software, validation, formal analysis, investigation, visualization. All authors have read and agreed to the published version of the manuscript.

## Funding

The authors have nothing to report.

## Disclosure

This article does not use the artificial intelligence (AI) generator to generate text.

## Ethics Statement

This study was reviewed and approved by the Ethical Committee of Henan Provincial People's Hospital. All experiments were conducted in accordance with the Declaration of Helsinki and relevant guidelines. Written informed consent was obtained from each participant. The animal experiments were complied with the ARRIVE guidelines and performed in accordance with the National Institutes of Health Guide for the Care and Use of Laboratory Animals. The experiments were approved by the Institutional Animal Care and Use Committee of Hangzhou Hebei Yunjing Technology Co. Ltd.

## Consent

Written informed consent was obtained from each subject.

## Conflicts of Interest

The authors declare no conflicts of interest.

## Supporting information


**Table S1:** Baseline demographic and clinical characteristics of the renal tissue cohorts.
**Table S2:** Antibodies used in this study.
**Table S3:** Primers for RT‐qPCR.

## Data Availability

The data that support the findings of this study are available on request from the corresponding author. The data are not publicly available due to privacy or ethical restrictions.
