# Supplementary material for: The ELAVL1‐PLAUR‐suPAR Axis Exacerbates Diabetic Nephropathy by Promoting Podocyte Injury and Inflammation
Source: J Diabetes. 2026 Aug 5;18(8):e70254. doi: 10.1111/1753-0407.70254 (PMC13441531; doi:10.1111/1753-0407.70254)
Supplement: Supplementary file 1 [file JDB-18-e70254-s001.docx]

**Table S1. Baseline demographic and clinical characteristics of the renal tissue cohorts**

| **Variable** | **Normal renal tissue controls (n = 15)** | **DN patients (n = 23)** | **P value** |
| --- | --- | --- | --- |
| Age, years | 55.9 ± 7.4 | 57.8 ± 10.1 | 0.508 |
| Male sex, n (%) | 7 (46.7) | 13 (56.5) | 0.741 |
| Diabetes duration, years | — | 8.4 ± 5.0 | — |
| HbA1c, % | 5.0 ± 0.4 | 7.8 ± 1.5 | <0.001 |
| UACR, mg/g | 10.3 (7.1–14.8) | 212.4 (110.8–332.1) | <0.001 |
| eGFR, mL/min/1.73 m² | 91.7 ± 11.1 | 52.6 ± 10.5 | <0.001 |

Data are presented as mean ± standard deviation, median (interquartile range), or n (%), as appropriate. Normally distributed continuous variables were compared using an independent-samples Student’s t-test or Welch’s t-test when the assumption of equal variances was not met. UACR was compared using the Mann–Whitney U test, and sex distribution was compared using Fisher’s exact test. The normal renal tissue controls were patients undergoing nephrectomy for renal tumors; histologically normal renal parenchyma distant from the tumor was used for tissue-level analyses. DN, diabetic nephropathy; HbA1c, glycated hemoglobin; UACR, urinary albumin-to-creatinine ratio; eGFR, estimated glomerular filtration rate; —, not applicable.

**Table S2. Antibodies used in this study**

| Primary antibodies | Supplier | Product ID | Application and Dilution |
| --- | --- | --- | --- |
| ELAVL1 | Proteintech, Wuhan, China | 11910-1-AP | IF 1:100 WB 1:1000 |
| uPAR | Proteintech, Wuhan, China | 10286-1-AP | IF 1:200 WB 1:1000 |
| Podocin | Proteintech, Wuhan, China | 20384-1-AP | IF 1:500 WB 1:600 |
| WT-1 | Proteintech, Wuhan, China | 12609-1-AP | IHC 1:1000 |
| ZO-1 | Proteintech, Wuhan, China | 21773-1-AP | WB 1:10000 |
| Desmin | Proteintech, Wuhan, China | 16520-1-AP | WB 1:20000 |
| Nephrin | Proteintech, Wuhan, China | 22912-1-AP | WB 1:2000 |
| β-actin | Proteintech, Wuhan, China | 66009-1-Ig | WB 1:10000 |

Abbreviations: ELAVL1, embryonic lethal abnormal vision-like 1; uPAR, urokinase-type plasminogen activator receptor; WT-1, Wilms tumor 1; ZO-1, zonula occludens-1; IF, immunofluorescence; IHC, immunohistochemistry; WB, western blot.

**Table S3. Primers for RT-qPCR**

| Gene name | Primer Sequences（5′ - 3′） |
| --- | --- |
| *ELAVL1* (human) | Forward: TCGGGATAAAGTAGCAGGACAC |
|  | Reverse: AGCGTGTTGATCGCTCTCTC |
| *PLAUR* (human) | Forward: GAGAGAAGACGTGCAGGGAC |
|  | Reverse: GCCCCAAGAGGCTGGGA |
| *ACTB* (human) | Forward: GAAGACTACGAGCTGCCTGA |
|  | Reverse: CAGACAGCACTGTGTTGGCG |
| *Elavl1* (mouse) | Forward: CAAACGGTCAGAAGCAGA |
|  | Reverse: CACCAGAAATCCCACTCA |
| *Plaur* (mouse) | Forward: AAGCCTGCAATG CCGCTATC |
|  | Reverse: GGGTGTAGTTGCAACACTTCAGGA |
| *Actb* (mouse) | Forward: TGTTCCTACCCCCAATGTGTCCGTC |
|  | Reverse: CTGGTCCTCAGTGTAGCCCAAGATG |

Abbreviations: RT-qPCR, reverse transcription quantitative polymerase chain reaction; ELAVL1, embryonic lethal abnormal vision-like protein 1; PLAUR, plasminogen activator, urokinase receptor; ACTB, beta-actin.
